# Effects of Meadow Connectivity on Plant Diversity in China's Poyang Lake: New Perspective of Effective Biodiversity Conservation

**DOI:** 10.1002/ece3.74328

**Published:** 2026-09-06

**Authors:** Cheng Zhang, Wenbo Chen, Qiaoqin Liu

**Affiliations:** ^1^ School of Environmental Science and Engineering Zhejiang University of Water Resources and Electric Power Hangzhou China; ^2^ School of Surveying and Spatial Information Engineering East China University of Technology Nanchang China; ^3^ School of Emergency Technology and Management University of Emergency Management Sanhe China

**Keywords:** functional connectivity, habitat fragmentation, meadow, plant diversity, Poyang Lake, scale effect

## Abstract

The increasing global habitat fragmentation has led to a marked decline in landscape connectivity, posing a severe threat to biodiversity. However, our understanding of how landscape connectivity influences biodiversity, particularly for plant diversity, remains notably limited. Taking the meadow of Poyang Lake as a case and comprehensively considering the ecological processes of water level change and species dispersal, this study firstly applied the landscape pattern metrics and the graph‐theoretic connectivity metrics to measure the dynamics of meadow structural connectivity and functional connectivity after identifying the spatial distribution of meadow at different water levels. Then, the effects of meadow connectivity on plant diversity and its scale effects were revealed through the linear regression model (LRM), and the redundancy analysis (RDA) was used to explore the contrasted explanatory of meadow structural connectivity and functional connectivity to plant diversity patterns. The results showed that: (1) Meadow was inundated and divided by water and its shrinkage coexisted with fragmentation when the water level rose. While meadow emerged and spliced and its expansion co‐occurred with cohesion when the water level fell. (2) When the water level increased, the shape of patch simplified, the area of patch shrank, the density of patch reduced, the aggregation of patch decreased and the meadow structural connectivity decreased progressively. Meanwhile, the number of components rose, the possibility of connectivity decreased and the meadow functional connectivity reduced dramatically. (3) Higher meadow connectivity not only led to greater plant species richness (α diversity) at the landscape scale but also increased plant community similarity (i.e., low β diversity) at the patch scale. Functional connectivity explains plant diversity patterns better than structural connectivity. This thesis proposes a new perspective of landscape connectivity for biodiversity conservation and landscape pattern optimization in lake areas.

## Graphical abstract

Higher meadow connectivity leads to greater plant species richness but also increases plant community similarity. Functional connectivity explains plant diversity patterns better than structural connectivity.

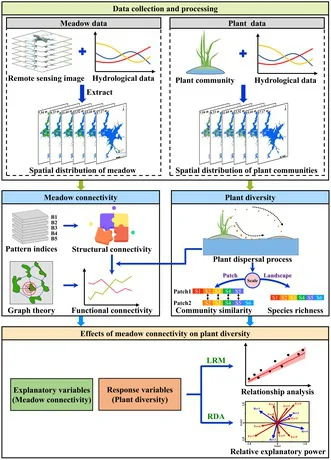

## 1 Introduction

Biodiversity conservation has long been a global priority. Accelerated industrialization and urbanization have intensified human disturbance to natural habitats, leading to progressively severe habitat shrinkage and fragmentation (Salmona et al. 2017). Among them, habitat fragmentation reduces landscape connectivity, thereby constraining key species dispersal processes such as plant pollination, seed dispersal, and wildlife migration, and ultimately threatening biodiversity (Yuan et al. 2024). As such, it has emerged as a central theme in research on the biological impacts of global change (Rios et al. 2021). Against the backdrop of escalating global habitat fragmentation, analyzing the spatiotemporal dynamics of landscape connectivity and elucidating its impacts on biodiversity are essential for restoring ecological processes and sustaining regional biodiversity (Uroy et al. 2019).

Landscape connectivity is considered to be the extent to which a landscape promotes or hinders the dispersal of organisms or certain ecological processes between habitat patches (Tischendorf and Fahrig 2000). It can be divided into landscape structural connectivity and functional connectivity (Goodwin 2003). Landscape structural connectivity is defined as the actual connectivity that only measures the geometric connection of habitat patches in physical structure rather than considering ecological processes (McGuire et al. 2016). Landscape functional connectivity refers to the potential connectivity simulated by incorporating landscape physical structure with species dispersal processes (Vogt et al. 2009). Functional connectivity represents the dispersal capacity of species among habitat patches and reflects the behavioral response of species to landscape structure, which means that it is more ecologically significant than structural connectivity (Karisto and Kisdi 2019). Currently, numerous studies on landscape connectivity emphasize functional connectivity, but the scientific representation and effective integration of ecological processes remain underdeveloped and require further methodological advancement (Gupta et al. 2019). In recent decades, several quantitative methods for assessing landscape connectivity have been developed, including landscape pattern metrics, circuit theory, the least‐cost model, and graph‐theoretic approaches (Foltête 2019). Landscape pattern metrics can effectively capture landscape spatial configuration, yet they poorly represent underlying ecological processes (Li et al. 2011). Some of these metrics that can reflect the physical connectivity of patches are commonly employed to quantify landscape structural connectivity (Buelow et al. 2017). The graph‐theoretic approach enables robust integration of landscape pattern and ecological processes (Zuecco et al. 2019). The graph‐theoretic connectivity metrics are widely used to model landscape functional connectivity and have been applied across diverse contexts, including biodiversity conservation and landscape planning (Iezzi et al. 2022; Yemshanov et al. 2025).

Since the beginning of the twenty‐first century, biodiversity in fragmented habitats has been intensively studied from multiple perspectives, including genetic isolation, habitat suitability, and habitat availability (Liu et al. 2019; Sitotaw et al. 2025). Notably, the effects of landscape connectivity on biodiversity have been investigated through experimental approaches by tracking the actual movement and spatial distribution of organisms among habitat patches (Inclan et al. 2014; Lino et al. 2019), or coupled with correlative approaches using quantitative indices (Hearn et al. 2019; Brauer and Beheregaray 2020). However, these studies have predominantly focused on animal taxa, yet our consensus about the effects of landscape connectivity on plant diversity remains limited. Moreover, the relative contributions of landscape structural connectivity versus functional connectivity to plant diversity patterns remain unclear and highly controversial. It is well known that biodiversity patterns exhibit scale dependence and vary across spatial scales of investigation (Burrows et al. 2019). Traditionally, local‐scale diversity has been quantified as the species richness observed within individual sampling plots in the focal habitat. At present, the effects of landscape pattern on biodiversity is increasingly analyzed from the perspective of landscape ecology, giving rise to two additional biodiversity scales. One is the patch‐scale diversity, which cares the species richness recorded across all sampling sites within a single habitat patch (Forsyth and Gilbert 2020). The other is the landscape‐scale diversity, which emphasizes the total species richness observed across all patches within the whole landscape (Pavlacky et al. 2022). Currently, many researchers quantify local‐scale biodiversity using diversity indices such as the Mar‐galef, Simpson, Pielou, and Shannon‐Wiener indices (Rowland et al. 2020). In contrast, studies addressing patch‐scale and landscape‐scale remain comparatively scarce. Consequently, this study explicitly examines how landscape connectivity differentially influences plant diversity across these two spatial scales.

**FIGURE 1:**
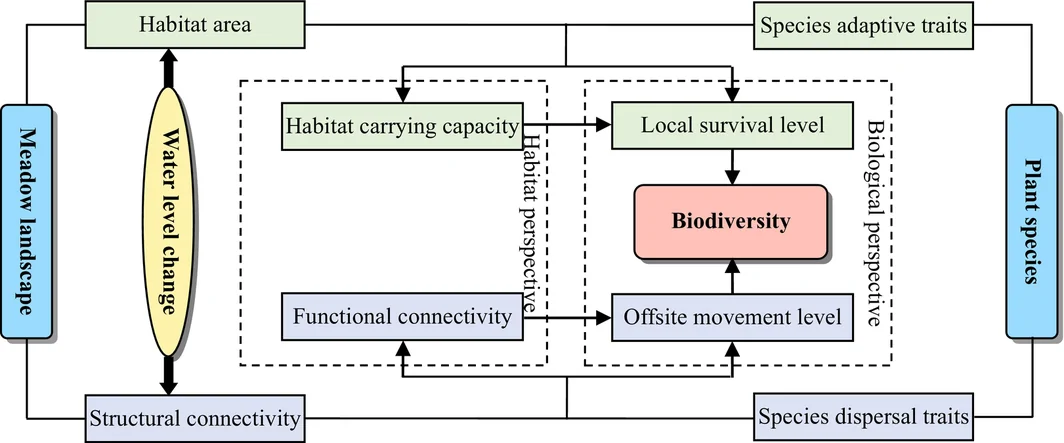
Mechanisms by which meadow landscape affects plant diversity in Poyang Lake.

Poyang Lake is recognized as one of the most ecologically significant wetland ecosystems due to its critical role in biodiversity conservation (Sun et al. 2020). Meadow is a characteristic vegetation landscape that emerges following seasonal water level recession. In recent years, meadows have been the subject of intensive research by numerous scholars. Remote sensing data were used to identify the spatiotemporal pattern of emergent meadow under contrasting hydrological conditions (Yang et al. 2016). Plant community development and its response to water level fluctuations have been investigated through integrated field surveys and controlled indoor experiments (Feng et al. 2020). However, these studies have primarily focused on the spatial configuration of meadow and its influence on plant community distribution across hydrological regimes. In contrast, the role of meadow connectivity in shaping plant diversity has yet to be revealed by considering ecological processes. In Poyang Lake, water level change and species dispersal represent two typical ecological processes (Zhang, Chen, He, et al. 2023). Under their combined influence, two distinct mechanisms of meadow landscape impacts on plant diversity were discovered (Figure 1) (Zhang, Chen, Huang, et al. 2023; Tan and Jiang 2016; Fu et al. 2021). For one thing, water level fluctuations drive the cyclical shrinkage and expansion of meadow, thereby altering both its total area and carrying capacity for plant populations, directly influencing plant species persistence within meadow patches and, consequently, plant diversity. For another, water level fluctuations induce the cyclical cohesion and division of meadow, thereby modifying their structural connectivity. Species dispersal capacity then determines whether plant propagules can traverse these structurally connected patches, which result in a differentiated meadow functional connectivity to different plant species, ultimately shaping plant diversity.

Thus, our aim is to: (1) measure the dynamics of meadow structural connectivity and functional connectivity after identifying the spatial distribution of meadow at different water levels; (2) reveal the effects of meadow connectivity on plant diversity and its scale effects, and further explore the contrasted explanatory of meadow structural connectivity and functional connectivity to plant diversity patterns. We hope this thesis can propose a new perspective of landscape connectivity for biodiversity conservation and landscape pattern optimization in lake areas.

## 2 Materials and Methods

### 2.1 Study Area

**FIGURE 2:**
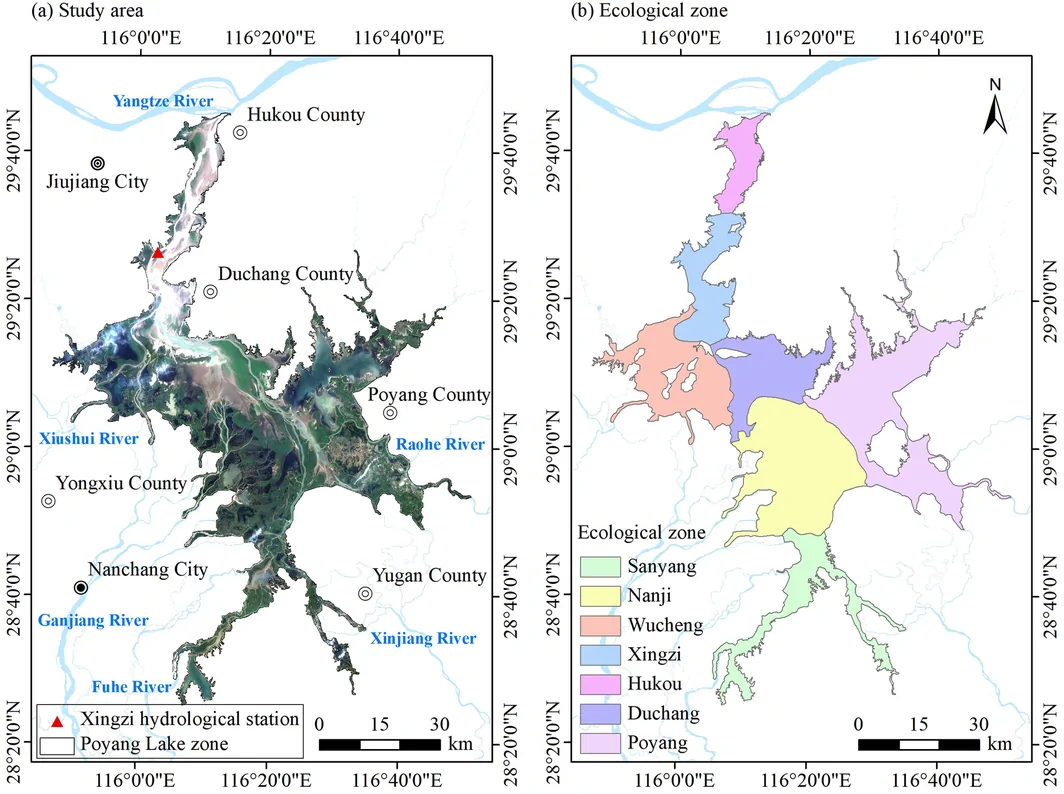
Spatial location of the study area and its ecological zone.

Our study was conducted in Poyang Lake, a typical freshwater lake located on the south bank of the middle and lower reaches of the Yangtze River in northern Jiangxi Province. With an area of approximately 3197.39 km^2^, the lake spans latitudes from 28°22′ to 29°45′N and longitudes from 115°47′ to 116°45′E (Figure 2). The region experiences a subtropical monsoon climate, characterized by a mean annual temperature ranging from 16.5°C to 17.8°C and mean annual precipitation ranging from 1358 to 1823 mm. As a unique through‐flow lake, Poyang Lake receives inflows from the Xiushui River, Ganjiang River, Xinjiang River, Raohe River, and Fuhe River before discharging into the Yangtze River (Yang et al. 2023). As a typical seasonal lake, it exhibits a flood season from April to September and a dry season from October to March (Tian et al. 2023). During the flood season, the meadow is inundated by water. During the dry season, it expands significantly and supports diverse plant communities (Shang and Chen 2024). Human disturbance to the meadow has been markedly reduced since the implementation of the grazing prohibition policy in 2008, thus providing an ideal experimental setting for this study.

The ecological zones of Poyang Lake are expected to exhibit significant differences in meadow connectivity and plant diversity. For one thing, the meadow connectivity in each ecological zone changes significantly with water level; that is, the meadow patches show obvious periodic inundation and emergence, and there is a certain elevation variation between meadow patches. For another, the plant diversity in each ecological zone varies dramatically with water level; that is, the plant community presents obvious heterogeneity in terms of composition, distribution, and diversity. Based on these principles, Poyang Lake was delineated into seven ecological zones: Hukou, Xingzi, Wucheng, Duchang, Poyang, Nanji, and Sanyang (Table 1).

**TABLE 1:** Characteristics of the ecological zones of Poyang Lake.

Ecological zone	Area (km^2^)	Area ratio (%)	Description
Hukou	165.01	5.16	The water area from Pingfeng to Hukou
Xingzi	289.58	9.06	The water area from Songmen Mountain to Pingfeng
Wucheng	461.25	14.43	The water area south of Songmen Mountain
Duchang	368.77	11.53	The north branch of Ganjiang River and the delta of Xiushui River
Poyang	810.60	25.35	Estuary delta of middle and south branches of Ganjiang River
Nanji	766.35	23.97	Estuary delta of Xinjiang River, Rao River and Tongjin River
Sanyang	335.83	10.50	Floodplain wet area of Fuhe River and Xinjiang River

### 2.2 Data Source and Processing

#### 2.2.1 Plant Community Data

Water level data at the Xingzi hydrological station during the dry season from 2000 to 2020, obtained from the Jiangxi Provincial Water Resources Department, indicate that Poyang Lake reached its lowest water level of 7.12 m on February 4, 2004, and its highest water level of 16.36 m on October 1, 2014. Using 7.12 m and 16.36 m as bounding thresholds and applying an approximate step size of 2 m, five water level gradients were established. Field surveys of plant communities were conducted by the method of combining sample sites with sample plots. A sample grid with a resolution of 1 × 1 km was established across the entire study area. Grid cells with meadow area more than 50% were selected as sample sites. Within each sample site, five sample plots were systematically deployed at the four corners and the geometric center according to the distribution of plant community. Plant community surveys were conducted respectively from December 1, 2007 to January 31, 2008 with the average water level of 7.63 m and 600 sample plots, October 15 to November 29, 2019 with the average water level of 9.44 m and 500 sample plots, October 11 to November 25, 2010 with the average water level of 11.47 m and 400 sample plots, October 1 to November 19, 2001 with the average water level of 13.28 m and 300 sample plots, and October 1 to 10, 2010 with the average water level of 15.29 m and 200 sample plots. Furthermore, remote sensing image indicated that meadow areas were fully inundated when the water level exceeded 18 m. We also found that the remote sensing image of Poyang Lake with high water level usually imaged in the flood season. Field surveys of plant communities were conducted from July 14 to August 2, 2007 with average water level of 17.10 m and 100 sample plots in order to comprehensively capture the dynamics of plant community across the full observed water level gradient.

#### 2.2.2 Remote Sensing Image Data

Six Landsat TM/ETM+ images with the imaging time and water levels closely matching the time period and mean water level of the field survey of plant communities were selected from the geospatial data cloud (https://www.gscloud.cn/) (Table 2). The cloud cover of these images is all less than 0.5%. The Landsat_gapfill plugin was applied to mitigate striping artifacts induced by the Scan Line Corrector (SLC) failure. Geometric correction and orthorectification were performed using a 1:50,000 digital topographic map of the Poyang Lake, projecting all images to the transverse Mercator coordinate system. A Landsat7/ETM+ image acquired on January 1, 2008 served as the radiometric reference; histogram matching was applied to all other images to harmonize cross‐sensor radiometric characteristics. Subsequently, all images underwent radiometric calibration, atmospheric correction, mosaicking, and cropping. Finally, false‐color composites were generated in ENVI 5.4 software using a band combination of 5:4:3.

**TABLE 2:** Information of remote sensing image data.

Order number	Image acquired date	Sensor	Water level (m)
1	1‐1‐2008	Landsat 7/ETM+	7.54
2	15‐11‐2019	Landsat 7/ETM+	9.33
3	6‐11‐2010	Landsat 7/ETM+	11.64
4	20‐10‐2001	Landsat 5/TM	13.52
5	5‐10‐2010	Landsat 7/ETM+	15.44
6	25‐7‐2007	Landsat 7/ETM+	17.17

### 2.3 Methods

**FIGURE 3:**
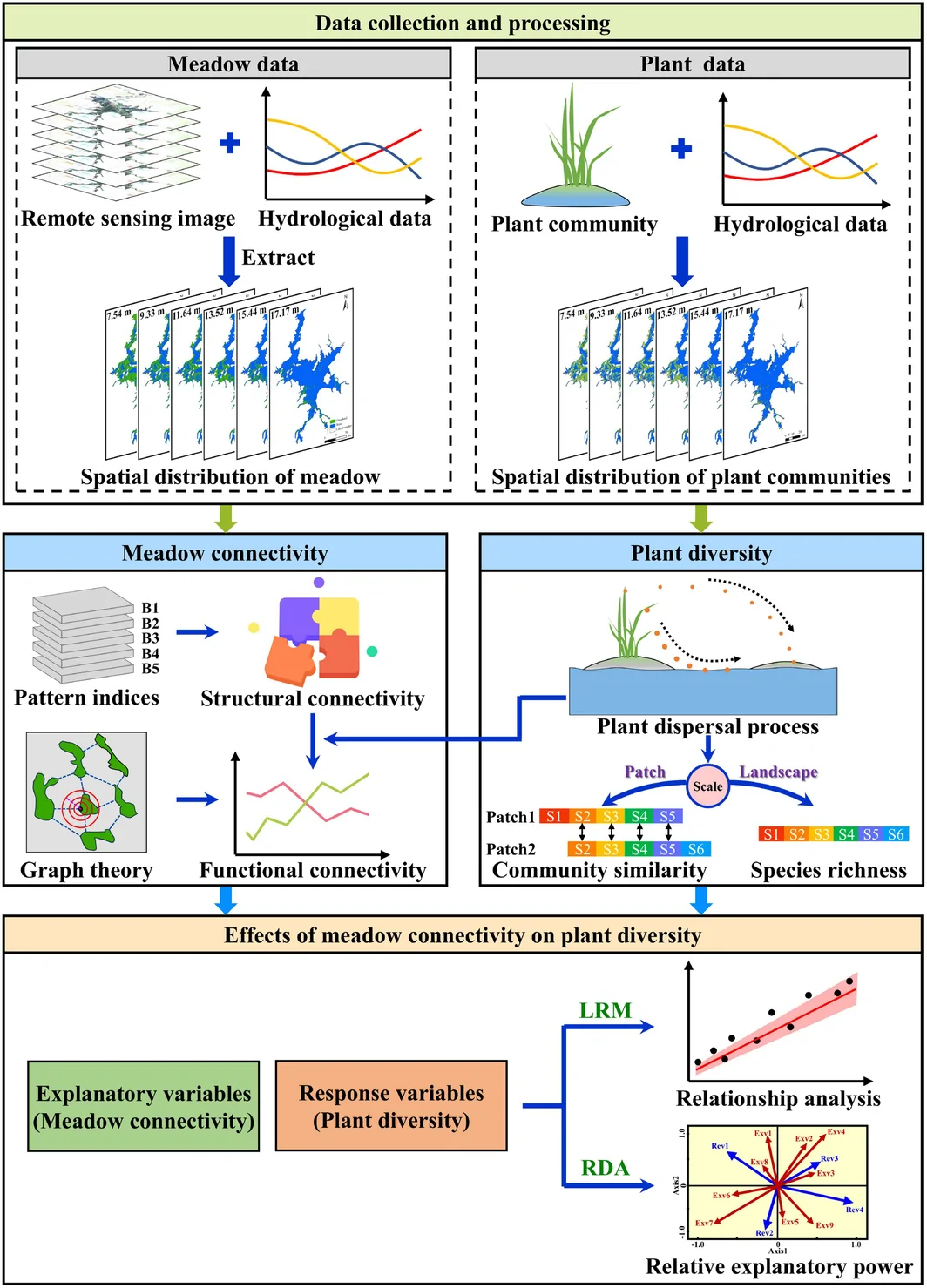
The study framework.

Firstly, with the help of water level data and Landsat images from 2000 to 2020, we identified the distribution pattern of meadow at different water levels. Then, meadow structural connectivity at different water levels was assessed using landscape pattern metrics. We also simulated meadow functional connectivity at different water levels by means of the graph‐based connectivity metrics through considering species dispersal process. Finally, linear regression model (LRM) was employed to evaluate the influence of meadow connectivity on plant diversity and its scale effects, and redundancy analysis (RDA) was subsequently applied to partition the relative explanatory contributions of structural versus functional connectivity to plant diversity patterns. The study framework was shown in Figure 3.

#### 2.3.1 Explanatory Variables: Meadow Structural and Functional Connectivity

Landscape pattern metrics can accurately express the structural composition and spatial configuration of landscape elements, and quantify the connectivity of landscape elements in spatial structure (Reulier et al. 2019). We selected landscape pattern metrics that can reflect the physical connection of patches from four complementary informational dimensions to measure meadow structural connectivity at the landscape scale. They include the following variables: largest patch index (LPI) and mean patch area (MPA) reflecting the characteristics of patch area, perimeter‐area fractal dimension (PAFRAC) and area weighted mean shape index (AWMSI) reflecting the characteristics of patch shape, edge density (ED) and patch density (PD) reflecting the characteristics of patch density, cohesion (COHESION) and division (DIVISION) reflecting the characteristics of patch aggregation. Person correlation analysis and significance test were conducted to detect multicollinearity among the landscape indices (Figures S1, S2) and thus six minimally redundant indices were retained for subsequent modeling (LPI, AWMSI, PAFRAC, ED, PD and COHESION) (Table S1). LPI quantifies the dominance of large patches and thus reflects landscape structural connectivity through patch size distribution. AWMSI measures shape complexity and indicates how irregular patch boundaries influence landscape structural connectivity. PAERAC characterizes landscape heterogeneity and serves as an indicator of cumulative disturbance from both natural processes and anthropogenic activities. ED and PD jointly quantify landscape fragmentation. ED reflects edge length per unit area, while PD represents the number of patches per unit area. COHESION indexes the degree of spatial aggregation among patches, with higher values indicating greater clumping and stronger structural continuity. All metrics were computed using Fragstats 4.2 software (McGarigal et al. 2012).

The graph theoretical approach integrates landscape spatial structure with species dispersal capacity to simulate ecologically meaningful functional connections among landscape elements (Reza et al. 2022). We selected the graph‐based connectivity indices from two complementary informational dimensions to quantify meadow functional connectivity at the landscape scale. They include the following variables: number of components (NC), flux index (F), number of links (NL), and harary index (H) reflecting the overall connectivity, area‐weighted flux (AWF), probability of connectivity (PC), and integral index of connectivity (IIC) reflecting the possible connectivity. Person correlation analysis was applied to eliminate redundancy (Figures S1, S2) and three minimally correlated indices were retained for downstream modeling (NC, F, and PC) (Table S1). NC quantifies the number of functionally interconnected patch components and thus indicates the degree of landscape fragmentation. F measures the total flux of potential movement across all links in the network, serving as an aggregate indicator of overall functional connectivity. PC integrates inter‐patch dispersal probabilities weighted by habitat area, providing a probabilistic measure of landscape functional linkage. These indices were calculated by the software of Conefor Sensinode 2.6 (Saura and Torné 2009).

#### 2.3.2 The Setting of Dispersal Distance Thresholds

The setting of dispersal distance thresholds is fundamental to computing the graph‐based connectivity indices (Baldwin and Myers 2024). Landscape components are defined as the whole in which patches are functionally interconnected with each other (Yang et al. 2024). We observed that the number of meadow components declined to one at a dispersal distance of 20,000 m and remained invariant beyond this threshold, which means that all meadow patches have established functional linkage (Figure S3). Therefore, according to variations in the number of meadow components and adhering to the principle of reflecting the dynamics and heterogeneity of meadow functional connectivity across dispersal distances, we selected sixteen dispersal distance thresholds (50, 100, 200, 400, 600, 800, 1000, 1500, 2000, 2500, 3000, 5000, 8000, 12,000, 16,000, and 20,000 m) for assessing meadow functional connectivity.

#### 2.3.3 | Response Variables: Plant Diversity

Plant diversity was quantified using α diversity (i.e., species richness) at the landscape scale and β diversity at the patch scale. To assess β diversity, we calculated Local Contributors of Beta Diversity (LCBD, Legendre and De Cáceres 2013). LCBD represents comparative indicators specifying the degree by which each sampling unit contributes to β diversity compared to a site with an average species composition, thus assessing ecological uniqueness in terms of species composition for each sampling unit. We defined $Y={\left[y_{\mathrm{ij}}\right]}_{n\times p}$ as a data table containing the abundance values of j plant species observed in i meadow patch. The calculation formula of ${\text{LCBD}}_{i}$ is as follows

(1) $${\text{LCBD}}_{i}=\sum_{j=1}^{p}{\left(y_{\mathrm{ij}}-\bar{y_{j}}\right)}^{2}/\sum_{i=1}^{n}\sum_{j=1}^{p}{\left(y_{\mathrm{ij}}-\bar{y_{j}}\right)}^{2}\;\left(i=1,2,3,\cdots ,n\right)$$

Thus, β diversity can be examined by LCBD and its calculation formula is as follows

(2) $$\text{LCBD}=\frac{1}{n}\sum_{i=1}^{n}{\left({\text{LCBD}}_{i}-\bar{{\text{LCBD}}_{i}}\right)}^{2}$$

#### 2.3.4 Correlation Analysis

For one thing, linear regression model (LRM) was employed to quantify the relationships between meadow connectivity indices and α and β diversity and the scale effects. For another, meadow structural and functional connectivity indices were treated as explanatory variables, and plant diversity indices served as response variables. Firstly, gradient analysis was performed using detrended corresponded analysis (DCA). Based on the gradient length of each explanatory axis (GLA), the appropriate constrained ordination method was selected, that is, redundancy analysis (RDA) is used when GLA is less than 3, canonical correspondence analysis (CCA) is used when LGA is greater than 4, and both models can be used when LGA is between 3 and 4. Subsequently, RDA or CCA was performed using the Canoco 5.0 software to ordinate the relationships between meadow connectivity indices and plant diversity, and corresponding biplots were generated. Finally, Monte Carlo permutation tests were applied to the ordination results to statistically evaluate and compare the relative explanatory power of meadow structural versus functional connectivity in shaping plant diversity patterns.

## 3 Results

### 3.1 Spatial Distribution of Meadow

**FIGURE 4:**
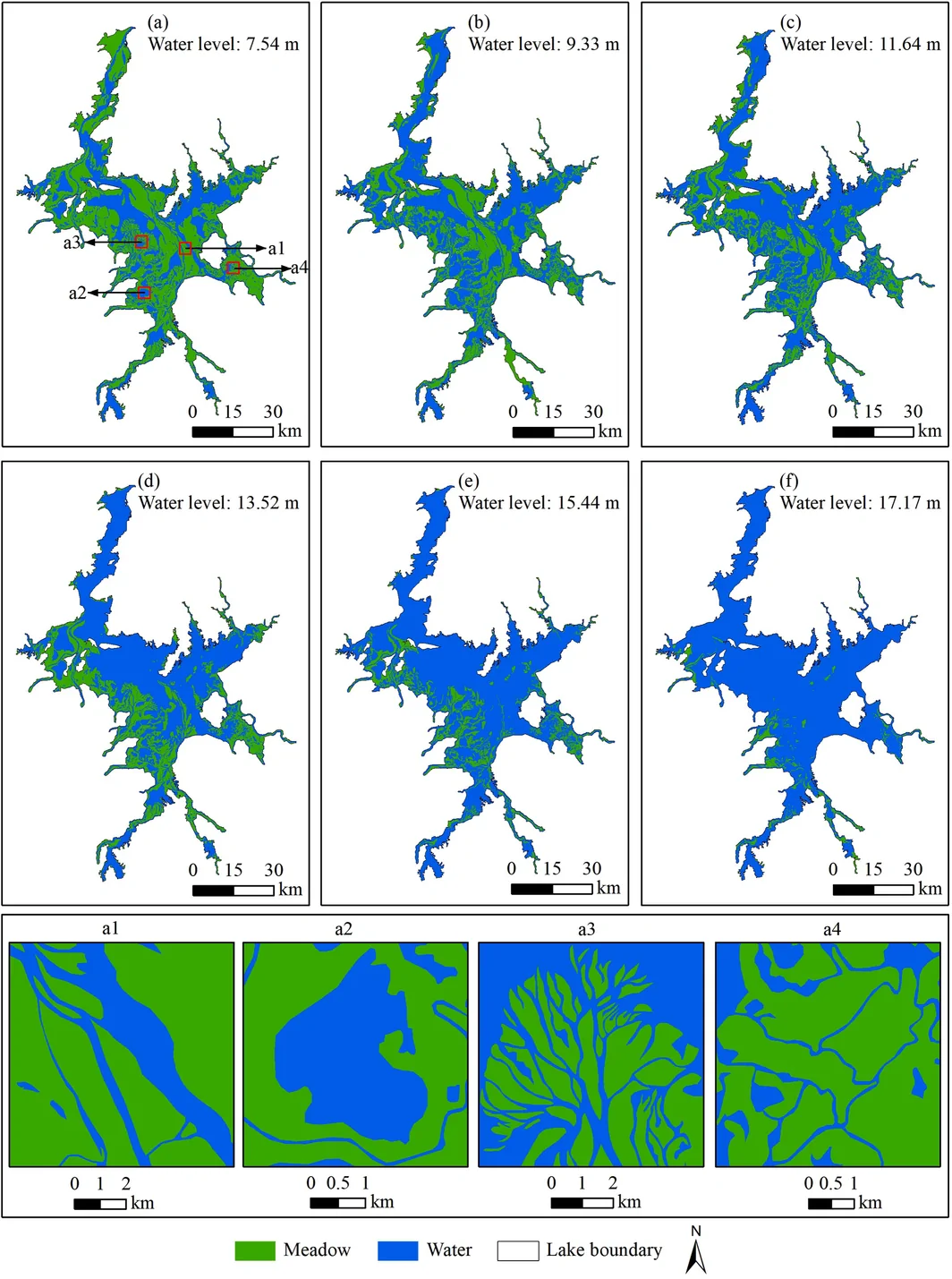
Spatial distribution of meadow at different water levels in Poyang Lake.

We identified the spatial distribution of meadow at different water levels using an integrated approach combining unsupervised classification, visual interpretation and NDWI threshold segmentation (Figure 4). Specifically, pixels with NDWI values greater than 0.3 were classified as water, whereas those with NDWI values less than 0.3 were classified as meadow. For each water level, we collected 120 field feature points along the meadow boundary and recorded their precise GPS coordinates. Classification accuracy was rigorously assessed by computing the root‐mean‐square error (RMSE) between GPS locations and the corresponding pixel centroids from the remote sensing interpretation, and results indicated that an overall meadow identification accuracy exceeded 95% at every water level. Additionally, the confusion matrix yielded a Kappa coefficient of 0.91, confirming excellent agreement between interpreted and reference data. At the landscape scale, meadow exhibited a regular spatial distribution aligned with the water level gradient. Three typical horizontal distribution structures were identified, which are the banded distribution structure along the water‐land intersection line (Figure 4a1), the circular distribution structure centered on the butterfly‐shaped lake (Figure 4a2), and the fan‐shaped distribution structure situated in the delta front (Figure 4a3). At the local scale, influenced by the fluctuation of small terrain and micro‐terrain, the meadow displayed a heterogeneous patchy distribution structure characterized by variable patch size and spatial arrangement (Figure 4a4).

**FIGURE 5:**
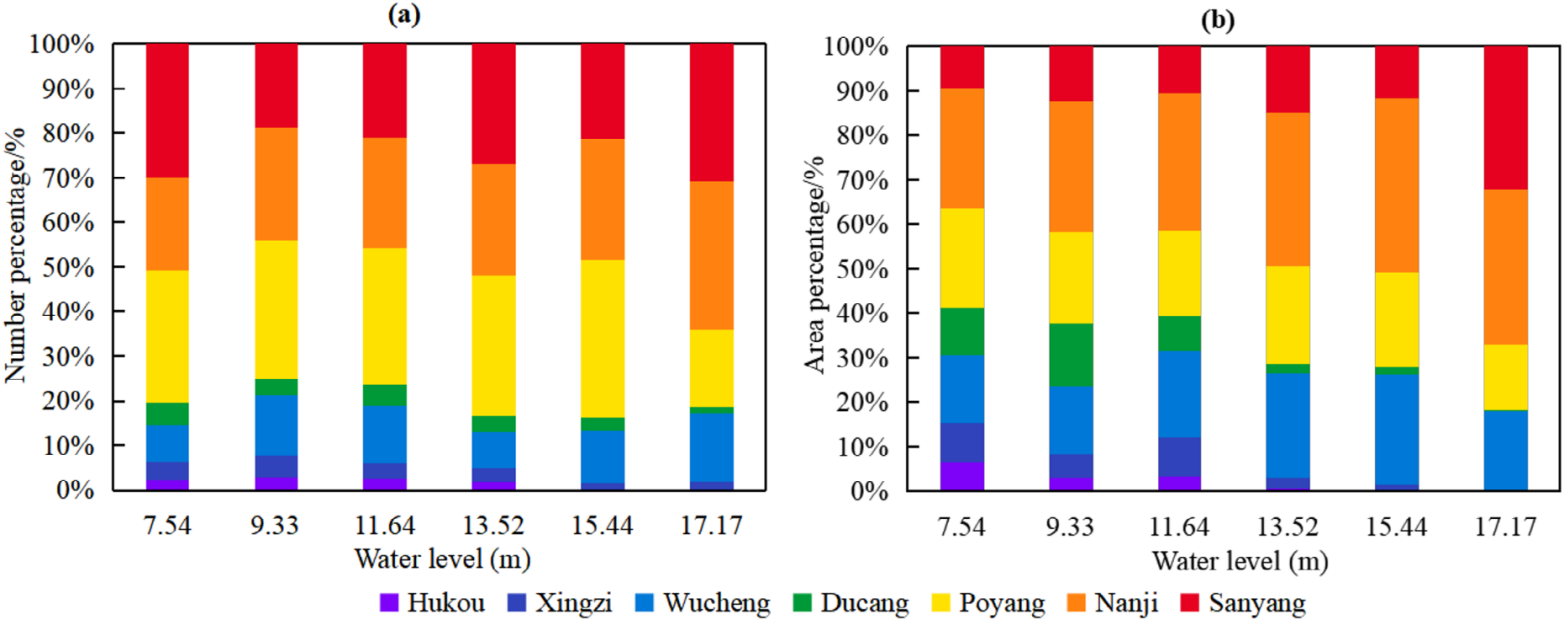
Number and area proportion of meadow patches for each ecological zone at different water levels.

The number and area of meadow patches were quantified separately for the entire lake and for each of the seven ecological zones at different water levels (Table 3 and Figure 5). Results revealed that water level fluctuations exerted a pronounced influence on meadow spatial distribution. Across the whole lake, the total number of meadow patches declined from 722 to 215 with a reduction of 70.22%, and the total area of meadow patches decreased from 1813.61 km^2^ to 182.36 km^2^ with a reduction of 89.94% as water level increased from 7.54 to 17.17 m. It demonstrated that the meadow was submerged by water and gradually shrank when water level rose. Concurrently, the mean patch area decreased from 2.512 km^2^ to 0.848 km^2^ with a reduction of 66.24% as water level rose from 7.54 to 17.17 m, which showed that the meadow was divided by water and progressively became fragmented as water level increased. Across the seven ecological zones, the number proportion of meadow patches was highest in Poyang, Nanji and Sanyang, intermediate in Wucheng, and lowest in Hukou, Xingzi and Duchang. Similarly, the area proportion of meadow patches was greatest in Nanji, followed by Poyang, Sanyang and Wucheng, with markedly lower contributions from Hukou, Xingzi and Duchang.

**TABLE 3:** Number and area of meadow patches for each ecological zone at different water levels.

Water level (m)	Meadow patch	Hukou	Xingzi	Wucheng	Duchang	Poyang	Nanji	Sanyang	Total
7.54	Number	16	30	58	38	214	149	217	722
	Area (km^2^)	119.33	157.79	277.50	192.11	407.19	488.17	171.52	1813.61
9.33	Number	23	39	109	32	249	206	152	810
	Area (km^2^)	40.37	70.52	196.54	185.35	267.91	380.17	163.25	1304.11
11.64	Number	18	24	86	34	210	169	144	685
	Area (km^2^)	34.80	96.04	208.73	81.42	205.71	332.21	114.37	1073.28
13.52	Number	9	15	40	19	155	124	133	495
	Area (km^2^)	6.60	22.55	226.49	20.29	209.57	334.46	141.55	961.51
15.44	Number	1	7	66	16	197	151	117	555
	Area (km^2^)	0.86	6.37	128.51	8.55	110.06	201.66	59.94	515.95
17.17	Number	0	4	33	3	37	72	66	215
	Area (km^2^)	0.00	0.51	32.11	0.46	26.94	63.87	58.47	182.36

### 3.2 Changes of Meadow Connectivity

#### 3.2.1 Meadow Structural Connectivity

**FIGURE 6:**
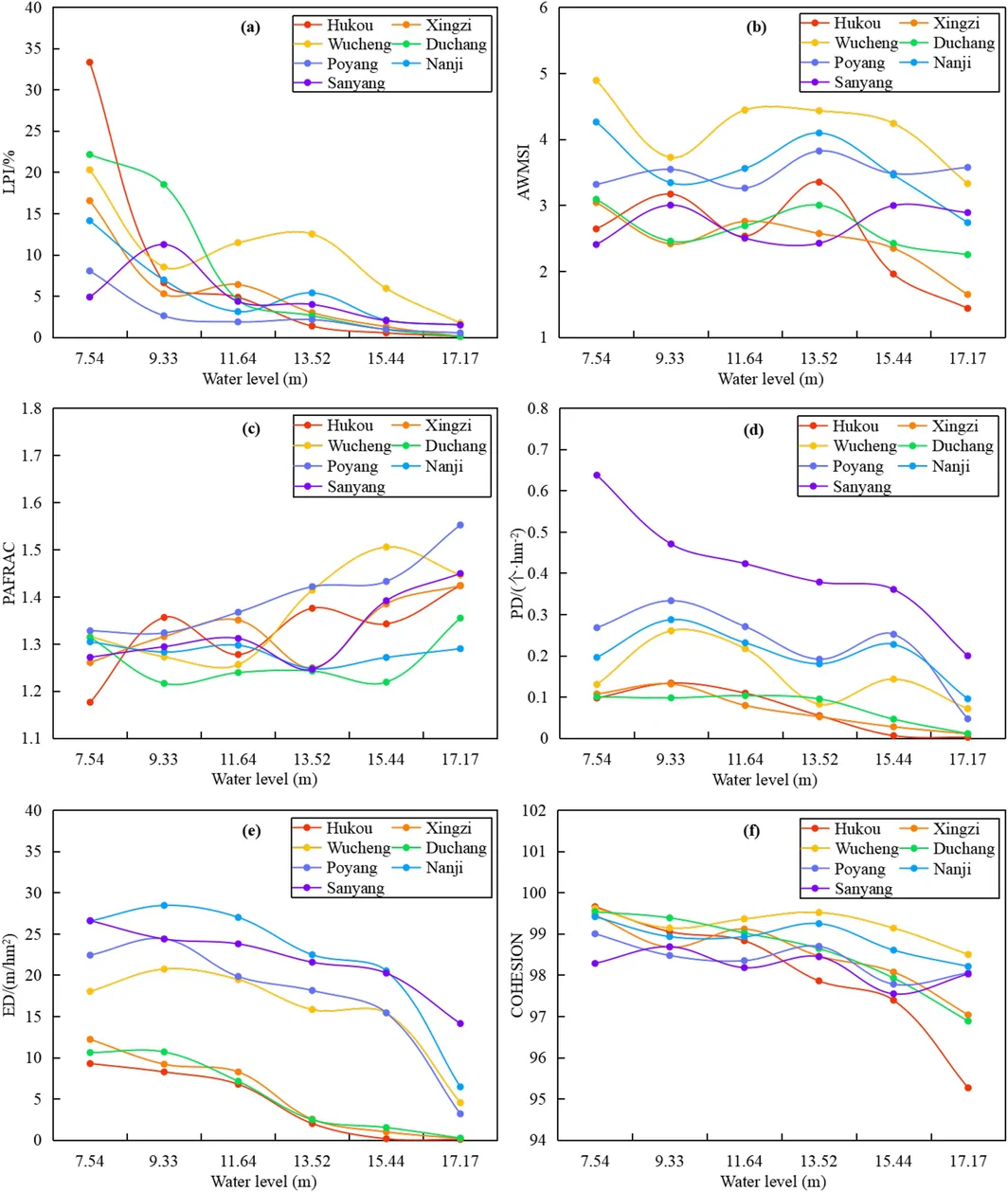
Meadow structural connectivity indices for each ecological zone at different water levels.

We calculated the indices of LPI, AWMSI, PAFRAC, PD, ED, COHESION to quantify meadow structural connectivity across all seven ecological zones under six water levels (Figure 6). Figure 6a shows that LPI declined consistently with increasing water level in every ecological zone, indicating progressive disintegration of large meadow patches into smaller, isolated units and concomitant habitat area reduction. Figure 6b reveals a generally decreasing, albeit fluctuating, trend in AWMSI, reflecting gradual simplification of meadow patch shape complexity. As can be seen from Figure 6c, PAFRAC decreased non‐monotonically with rising water level, indicating progressive intensification of structural disturbance driven by hydrological dynamics. Figure 6d,e show concurrent declines in PD and ED, signifying reduced patch density per unit area and diminished total edge length and indicating reduced spatial configuration complexity and advancing fragmentation. We can see from Figure 6f that COHESION presented a pronounced decline, confirming progressive spatial isolation of meadow patches and a substantial erosion of meadow structural connectivity.

#### 3.2.2 Meadow Functional Connectivity

**FIGURE 7:**
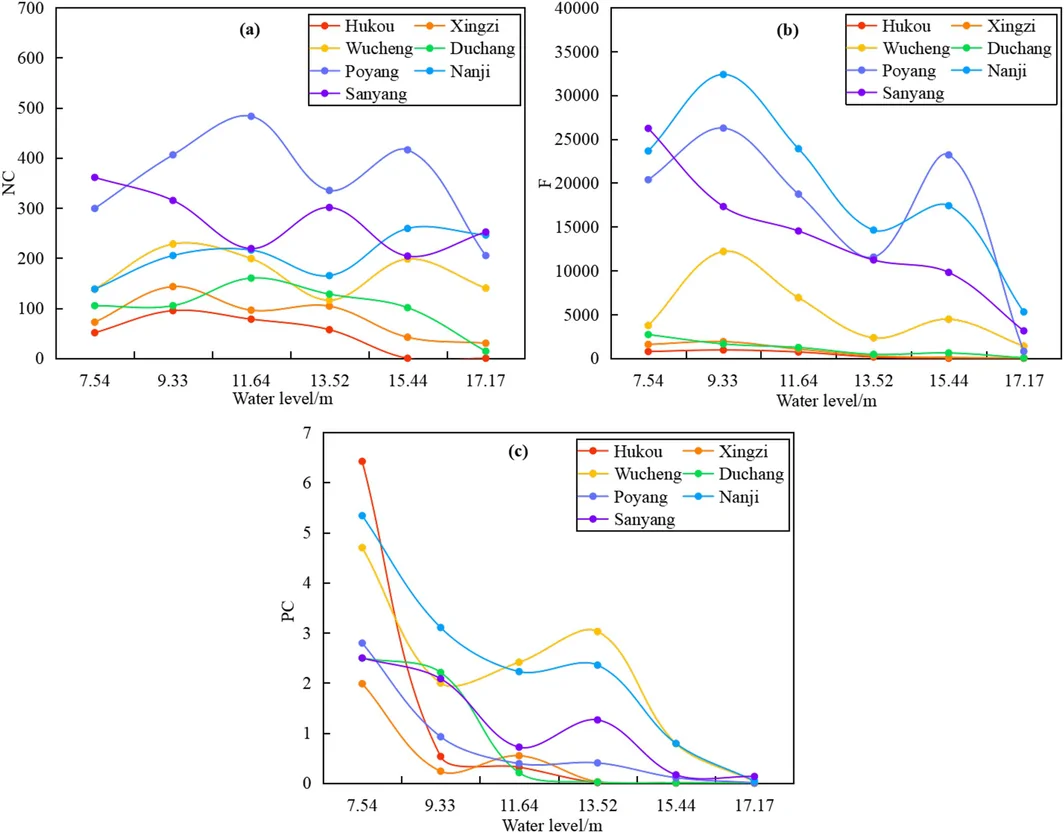
Meadow functional connectivity indices for each ecological zone at different water levels.

Under six water level scenarios, graph‐theoretic connectivity indices were calculated for seven ecological zones across sixteen dispersal distance thresholds. Because species with differing dispersal capacities respond independently to landscape pattern, the indices computed at each threshold were equally weighted and aggregated to quantify the dynamics of meadow functional connectivity under varying water levels (Figure 7).

We observed a gradual decline in NC with increasing water level across all seven ecological zones. On one hand, rising water levels led to submergence of meadow habitat by water bodies, reducing the total number of meadow patches and thereby decreasing NC. On the other, as the water level rise also fragmented the meadow landscape, increasing inter‐patch distances beyond the species dispersal thresholds and resulting in progressive isolation of previously connected meadow patches and their conversion into independent components. The dynamic of NC with water level indicates that the inundation of the water body on meadow is greater than its fragmentation. Spatially, at any given water level, Poyang, Nanji, and Sanyang consistently exhibited the highest NC values; Wucheng and Duchang ranked intermediate; and Hukou and Xingzi showed the lowest NC values. F showed a generally fluctuating downward trend with increasing water level across all ecological zones, indicating progressive fragmentation and spatial isolation of meadow patches. As inter‐patch distances increased beyond species‐specific dispersal thresholds, effective dispersal among patches became increasingly constrained, leading to a progressive decline in overall meadow functional connectivity. At any given water level, F values were highest in Poyang, Nanji, and Sanyang; intermediate in Wucheng; and lowest in Duchang, Hukou, and Xingzi. We can conclude from Table 3 that this spatial pattern strongly correlated with both patch area and patch number within each ecological zone. We can see that PC decreased significantly with rising water level and approached zero when at 17.17 m, indicating intensified meadow fragmentation and a progressive decline in potential functional connectivity. Spatially, Wucheng and Nanji exhibited the highest PC values, followed by Sanyang, and Poyang; Hukou, Xingzi, and Duchang showed comparatively lower values.

### 3.3 Characteristics of Plant Community

#### 3.3.1 Composition of Plant Communities

Vegetation classification remains methodologically complex and lacks a universally accepted standard currently. In China, wetland vegetation classification is currently conducted primarily according to the framework outlined in “Chinese Vegetation,” which integrates three core criteria of species composition, habitat characteristics, and community structural features (Wu et al. 2021). Based on this classification system and our field survey data, meadow vegetation in the Poyang Lake region was categorized into two vegetation types, five vegetation subtypes, and thirty‐eight formations (Table 4). Non‐zonal herbaceous marsh vegetation dominated the meadow landscape, with herbaceous species constituting the overwhelming majority of the flora. Dominant constructive species of plant communities exhibit broad global distributions, such as Form. *Carex* spp., Form. *Phragmites australis* , Form. *Polygonum* spp. Rare and endangered plant species were scarce. Field surveys identified thirty‐eight dominant plant formations, including five Form. *Hygropoion*, nine Form. *Emersiprata*, four Form. *Cyperus*, seventeen Form. *Mesopoion*, and one artificial vegetation.

**TABLE 4:** Classification of plant communities in Poyang Lake.

Vegetation type	Vegetation subtype	Formation
Herbaceous marsh vegetation	Hygropoion	*Phragmites australis, Glyceria leptolepis, Triarrhena lutarioriparia, Zizania caduciflora, Phalaris arundinacea*
	Emersiprata	*Leersia japonica, Arundinella hirta, Panicum bisulcatum, Imperata cylindrica, Echinochloa spp, Setaria spp, Cynodon dactylon, Eremochloa ophiuroides, Hemarthria altissima*
	Cyperus	*Carex spp, Eleocharis japonica, Cyperus rotundus, Fimbristylis dichotoma*
	Mesopoion	*Typha orientalis, Typha angustifolia, Potentilla limprichtii, Cardamine lyrata, Polygonum criopolitanum, Polygonum japonicum, Polygonum lapathifolium, Polygonum minus, Polygonum posumbu, Polygonum praetermissum, Polygonum hydropiper, Rumex dentatus, Artemisia selengensis, Artemisia lancea, Cotula amthemoides, Astragalus sinicus, Lobelia chinensis*
Artificial vegetation	Artificial forest	*Populus canadensis, Sapium sebiferum, Salix matsudana*

It can be seen from Table 5 that the plant community was dominated by Form. *Phragmites australis* , Form. *Triarrhena lutarioriparia*, Form. *Phalaris arundinacea* , Form. *Carex* spp., and Form. *Polygonum criopolitanum*. At the whole lake scale, Form. *Carex* spp. constituted the largest plant community. At the water level of 7.54 m, Form. *Carex* spp. occupied 48.36% of the total meadow area, followed by Form. *Phalaris arundinacea* , accounting for 11.58%. Form. *Polygonum criopolitanum* also played an important role, accounting for 11.26%. Water level fluctuations induced substantial changes in both the area and number of meadow patches supporting these plant communities. As water level increased from 7.54 m to 17.17 m, the total number of plant community patches declined from 1557 to 521 with a reduction of 66.54%, and the total area of plant community patches decreased from 1813.61 km^2^ to 182.36 km^2^ with a reduction of 89.94%.

**TABLE 5:** Number and area of meadow patches occupied by plant communities at different water levels.

Plant community	Patch	Water level (m)					
		7.54	9.33	11.64	13.52	15.44	17.17
*Phragmites australis*	Number	199	296	291	219	250	87
	Area (km^2^)	197.82	148.83	137.31	166.47	104.54	34.35
Glyceria leptolepis	Number	3	3	3	5	3	0
	Area (km^2^)	7.26	5.89	4.25	1.86	1.74	0.00
Triarrhena lutarioriparia	Number	114	174	165	184	164	52
	Area (km^2^)	98.57	82.80	78.19	89.23	55.21	15.00
*Zizania caduciflora*	Number	27	36	36	23	36	7
	Area (km^2^)	10.19	7.11	8.46	10.32	7.62	2.81
*Phalaris arundinacea*	Number	133	206	189	157	95	8
	Area (km^2^)	210.06	176.46	109.55	53.20	20.75	4.32
Emersiprata	Number	116	134	119	114	121	59
	Area (km^2^)	64.03	63.47	54.43	51.72	36.36	28.49
Carex spp	Number	695	1007	845	665	702	212
	Area (km^2^)	876.40	625.51	531.75	503.74	244.38	63.25
Eleocharis japonica	Number	3	3	3	3	2	0
	Area (km^2^)	3.99	2.68	2.12	1.52	0.60	0.00
*Cyperus rotundus*	Number	20	36	31	23	8	2
	Area (km^2^)	59.44	41.90	32.74	5.47	2.43	1.19
*Fimbristylis dichotoma*	Number	3	3	3	3	3	0
	Area (km^2^)	1.11	0.88	1.02	0.94	0.59	0
Polygonum criopolitanum	Number	86	127	95	39	12	3
	Area (km^2^)	204.16	84.23	53.98	6.33	2.79	0.08
*Polygonum hydropiper*	Number	59	57	23	36	11	17
	Area (km^2^)	25.25	25.10	12.02	19.12	5.21	8.04
Other mesopoion	Number	37	36	38	40	11	7
	Area (km^2^)	9.89	5.95	5.89	5.22	1.51	1.22
Artificial vegetation	Number	65	102	96	91	82	67
	Area (km^2^)	46.55	34.18	42.59	47.31	32.81	23.61
Sum	Number	1557	2217	1934	1599	1497	521
	Area (km^2^)	1813.61	1304.11	1073.28	961.51	515.95	182.36

#### 3.3.2 Spatial Distribution of Plant Communities

**FIGURE 8:**
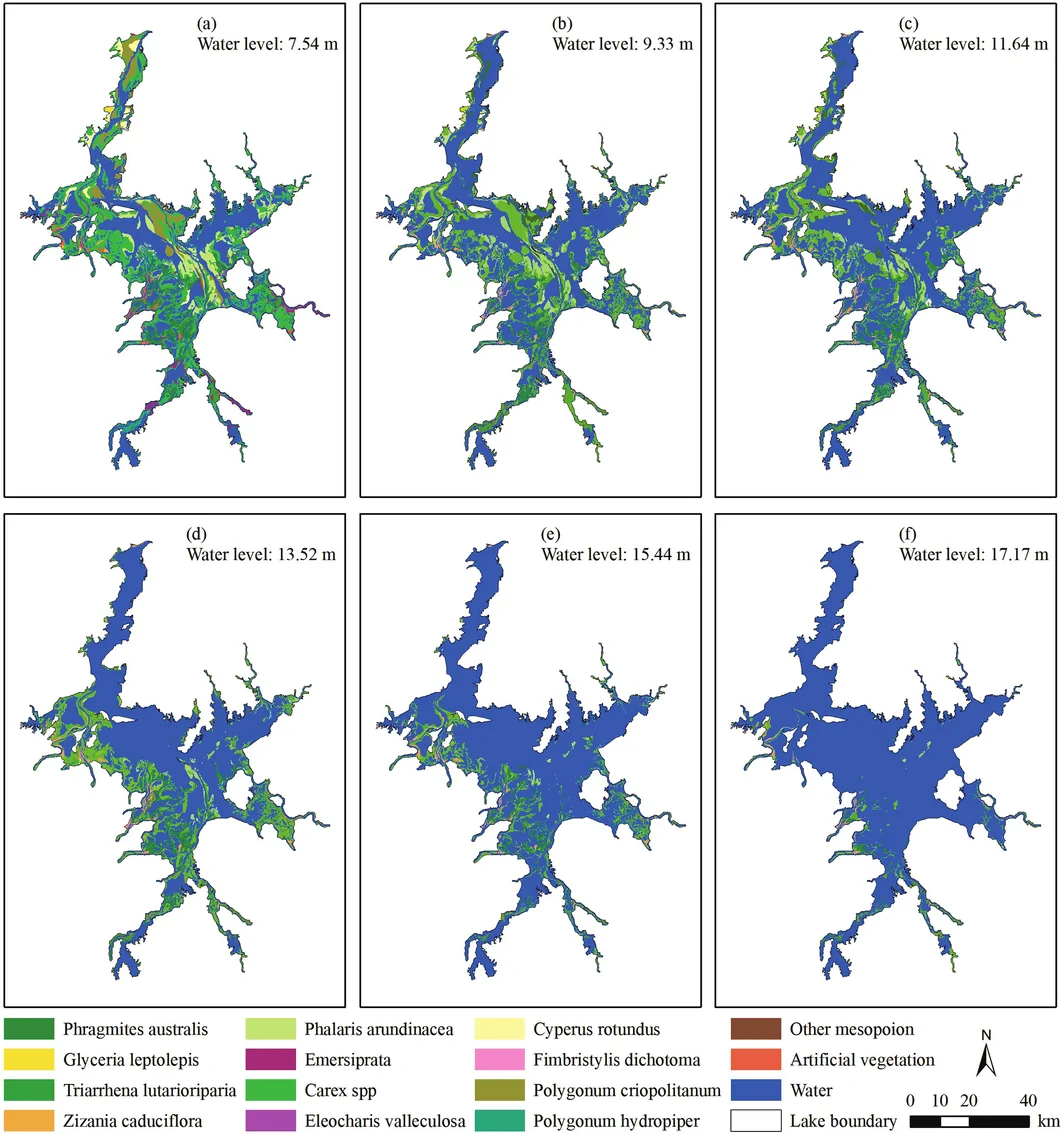
Spatial distribution of plant communities at different water levels in Poyang Lake.

The distribution patterns of plant communities were significantly influenced by water level fluctuations. Along the water level gradient, plant communities exhibited a regular spatial arrangement (Figure 8). At the whole lake scale, three distinct horizontal distribution structures were identified, namely the banded distribution structure along the river channel, the circular distribution structure centered on the seasonal butterfly‐shaped lake, and the fan‐shaped distribution structure located in the delta front. Concurrently, micro‐topographic variations drove elevation‐dependent patchiness in plant community composition. At fine scales, it manifested as the patchy mosaic of plant species. At intermediate scales, a complex formed by different plant communities, such as the patchy two‐component complex composed of Form. *Triarrhena lutarioriparia* and Form. *Carex* spp.

#### 3.3.3 Diversity of Plant Communities

**FIGURE 9:**
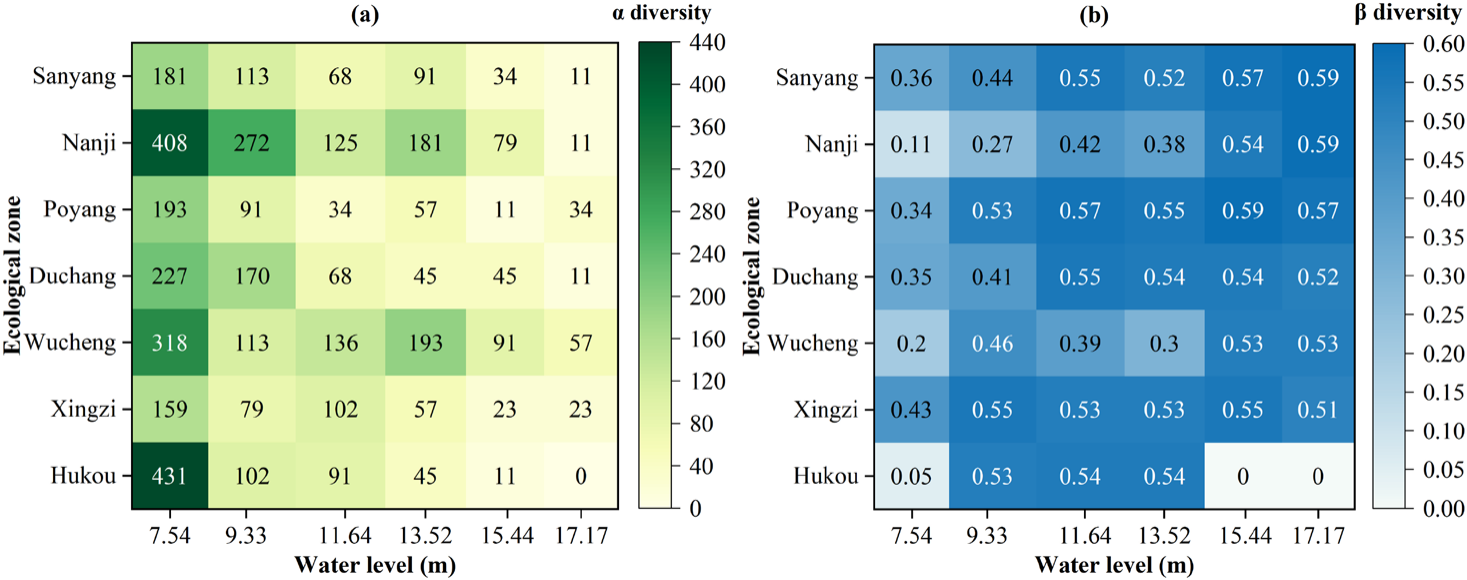
α and β diversity of plant communities for each ecological zone at different water levels.

Across the entire lake and at the α diversity level, field sampling recorded 431 plant species (Figure 9a). At the β diversity level, the LCBD values ranged from 0 to 0.59 (Figure 9b). The α diversity peaked across all seven ecological zones at the water level of 7.54 m, whereas the β diversity reached its minimum at this same threshold. With increasing water level, α diversity declined significantly, while β diversity increased monotonically. These findings indicate that elevated water levels reduce plant species richness at the landscape scale but concurrently decrease plant community similarity at the patch scale. Spatially, under identical water level gradients, the spatial variance of α diversity exceeded that of β diversity across the seven ecological zones, demonstrating stronger spatial differentiation in α diversity than in β diversity. Correlation analysis revealed that α diversity had a statistically significant negative relationship with β diversity (*p* < 0.05) (Figure S4).

### 3.4 Effects of Meadow Connectivity on Plant Diversity

#### 3.4.1 The Influence of Meadow Connectivity on α and β Diversity and Its Scale Effects

**FIGURE 10:**
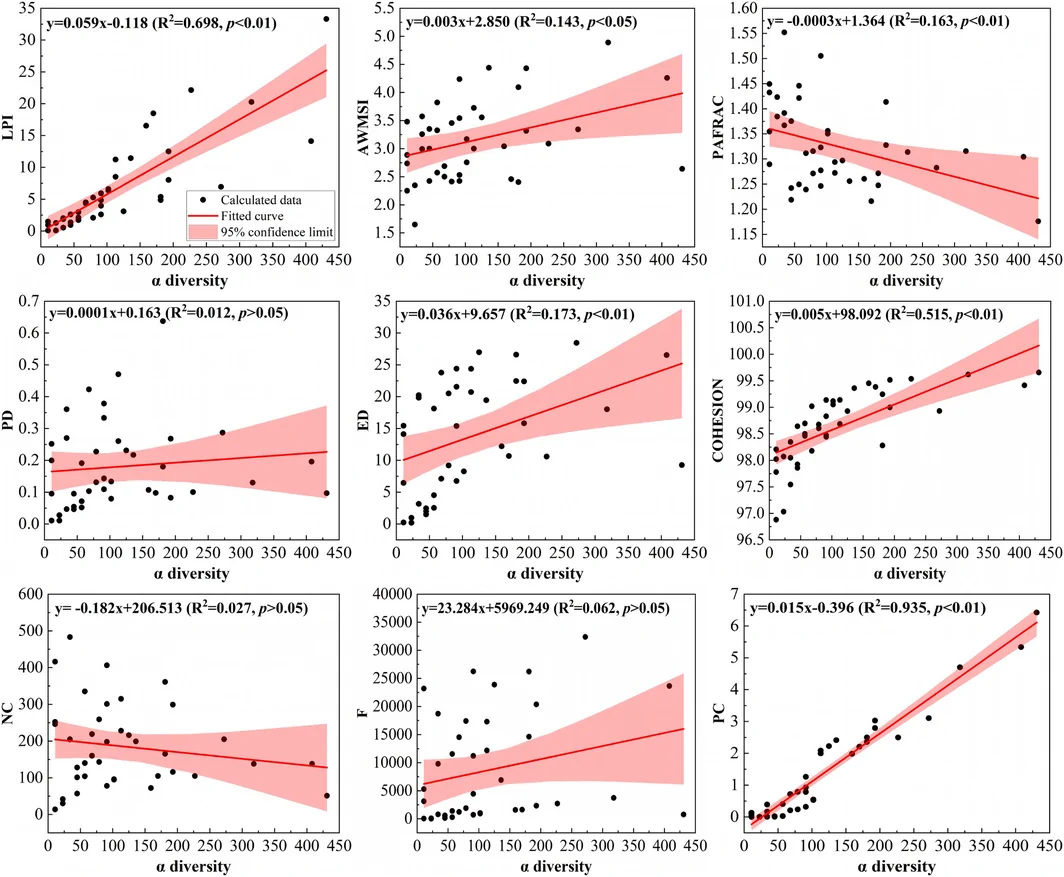
Linear regression relationships between α diversity and grassland connectivity indices.

**FIGURE 11:**
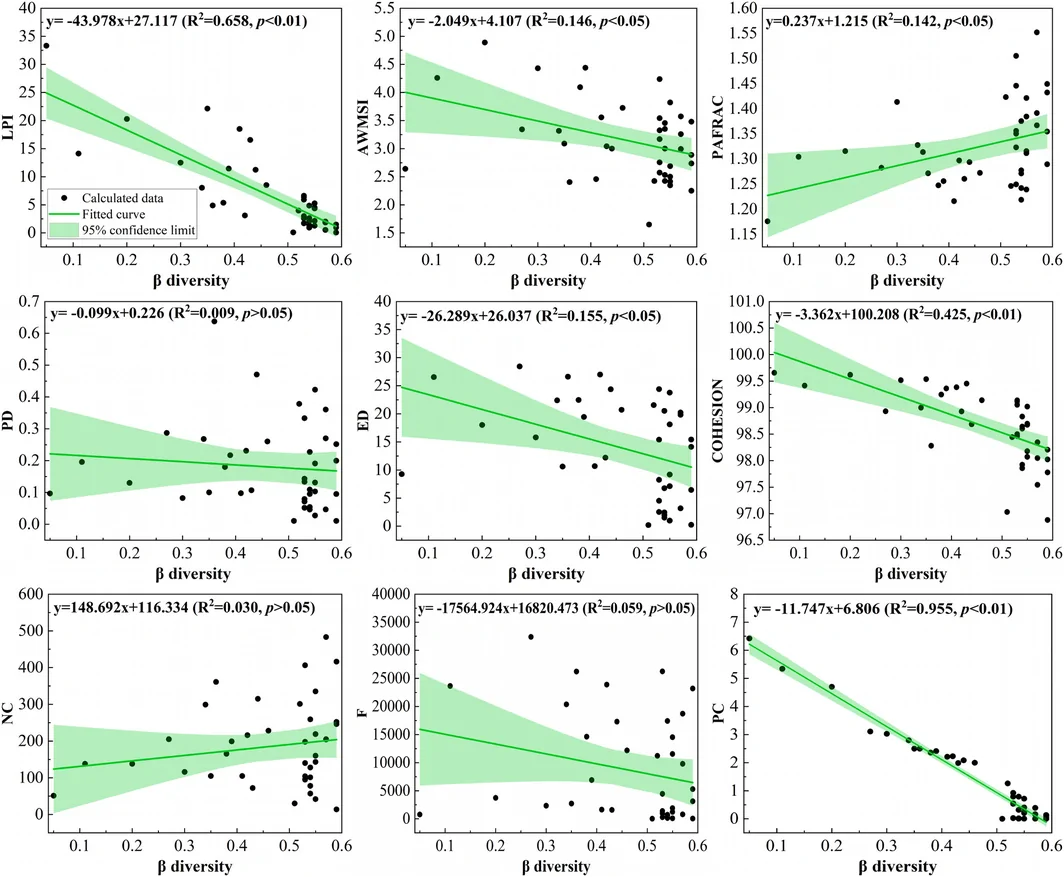
Linear regression relationships between β diversity and grassland connectivity indices.

A linear regression model (LRM) was employed to assess the relationships between meadow connectivity indices and both α diversity (Figure 10) and β diversity (Figure 11). Overall, meadow connectivity exerted a statistically significant influence on plant diversity. Higher meadow connectivity not only led to greater plant species richness (α diversity) at the landscape scale but also increased plant community similarity (i.e., low β diversity) at the patch scale. When distinguishing structural from functional connectivity, both metrics showed positive correlations with α diversity and negative correlations with β diversity, indicating a consistent directional effect of meadow connectivity on plant diversity patterns across scales.

Regarding individual landscape connectivity metrics and their characteristic dimensions, in addition to PD, all meadow structural connectivity indices (COHESION, ED, PD, PAFRAC, AWMSI and LPI) had significant relationships with both α diversity and β diversity. Specifically, α diversity showed positive correlations with COHESION, ED, AWMSI and LPI, but a negative correlation with PAFRAC. In contrast, β diversity displayed negative correlations with COHESION, ED, AWMSI and LPI, and a positive correlation with PAFRAC. Notably, COHESION and LPI exhibited the strongest associations with both diversity measures, indicating that patch area and aggregation rather than other structural metrics exerted the most substantial influence on plant diversity. Among meadow functional connectivity indices, F and NC showed no significant relationships with α and β diversity, whereas PC incorporating inter‐patch dispersal probability demonstrated statistically significant correlations with both. This underscores that plant diversity is closely linked to species dispersal capacity across the meadow landscape.

#### 3.4.2 The Contrasted Explanatory of Meadow Structural Connectivity and Functional Connectivity on Plant Diversity Patterns

**FIGURE 12:**
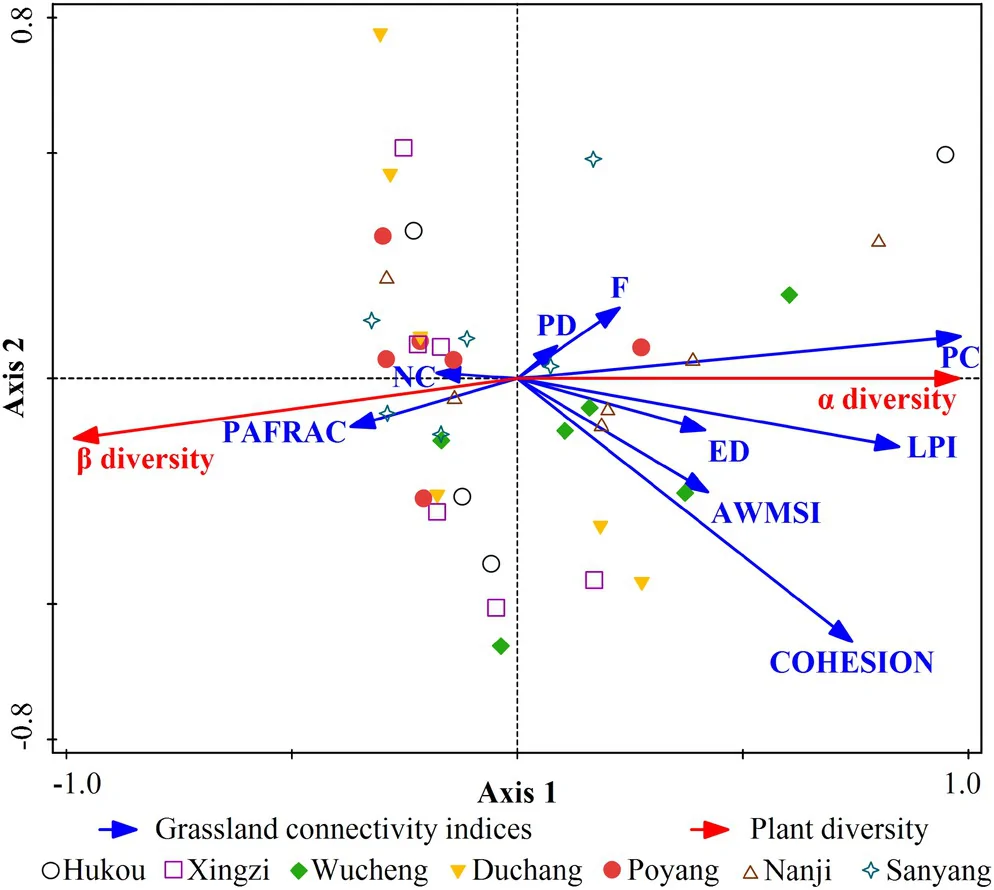
The coordination map of meadow connectivity indices and plant diversity.

Gradient analysis indicated a gradient length of 0.21, confirming that RDA was more appropriate than CCA for modeling species‐environment relationships in this dataset. Then, RDA was applied to analyze the associations between meadow connectivity indices and plant diversity (Figure 12), thereby disentangling the contrasted explanatory of meadow structural connectivity and functional connectivity on plant diversity patterns (Table 6). The RDA model for α diversity explained 86.2% of the variation (*R* ^2^ = 0.862, *p* < 0.05) and retained PC, NC, and COHESION as significant predictors at the landscape scale. The RDA model for β diversity accounted for 88.1% of the variation (*R* ^2^ = 0.881, *p* < 0.01) and selected PC, LPI, and ED as significant predictors at the patch scale. Notably, PC exhibited significantly greater explanatory power for both α diversity and β diversity than LPI, ED, and COHESION across both landscape scale and patch scale, indicating that functional connectivity explains plant diversity patterns better than structural connectivity.

**TABLE 6:** The RDA model testing the explanatory power of meadow connectivity indices to plant diversity.

Response variables	Explanatory variables	Explains (%)	Contribution (%)	Pseudo‐F	*p*	*R* ^2^
α diversity	PC	53.5	56.8	346	0.002**	86.2%
	NC	21.1	21.1	175	0.006**	
	COHESION	20.5	20.5	138	0.018*	
β diversity	PC	55.6	57.3	419	0.002**	88.1%
	ED	21.2	21.3	342	0.004**	
	LPI	20.7	20.7	295	0.010**	

*Note:* * and ** represent a significance level of 95% and 99% respectively.

## 4 Discussion

### 4.1 The Scale Dependence of Biodiversity Patterns

In landscape ecology, it is well established that biodiversity patterns are scale‐dependent and vary across spatial scales of investigation (Burrows et al. 2019). In this study, we examined how landscape connectivity influences plant diversity by considering plant species richness at the landscape scale and plant community similarity at the patch scale. Our results reveal contrasting effects of landscape connectivity on plant diversity when considering different scales (i.e., landscape and patch). Our key findings is that higher meadow connectivity not only led to greater plant species richness (α diversity) at the landscape scale but also increased plant community similarity (i.e., low β diversity) at the patch scale. At the landscape scale, high meadow connectivity likely facilitates seed dispersal among meadows, thereby expanding the effective habitat area available to plant species and reducing local extinction risk, and ultimately promoting plant species richness. At the patch scale, high meadow connectivity likely enhances propagule flux among patches, thereby promoting species exchange and homogenizing plant community composition. Our findings provide critical insights into how landscape connectivity differentially shapes plant diversity across scales, thereby improving understanding of plant species responses to landscape fragmentation and informing evidence‐based strategies for optimizing landscape configuration to conserve plant diversity.

### 4.2 Explanation of Functional Connectivity for Plant Diversity Patterns

Animal species actively disperse among habitat patches through individual movement (Sinsch 2014). The influence of landscape connectivity on animals primarily depends on their capacity to actually move between habitat patches (Osipova et al. 2019). In other words, animals respond most strongly to landscape structural connectivity. The actual connectivity for animals can be investigated by comparing the physical distance between habitat patches with the moving distance of organisms via field experiments (Xia et al. 2020). In this study, we essentially identified the contrasted explanatory factors of meadow structural connectivity and functional connectivity to plant diversity patterns, providing robust evidence that functional connectivity explains plant diversity patterns better than structural connectivity. Plant communities disperse passively among habitat patches via pollen, seeds, or biotic vectors (Teller et al. 2015). The influence of landscape connectivity on plants depends critically on the capacity of propagules to disperse effectively between habitat patches. Propagules may successfully reach other habitat patches if those patches are functionally connected even if structurally disconnected (Fink and Scheidegger 2018). In summary, landscape functional connectivity governs plant occurrence within habitat patches. For plants, functional connectivity can be quantified by comparing the physical distance between habitat patches with the potential dispersal distance of propagules using indices or mechanistic models (Plue et al. 2019).

### 4.3 Insights Into the Effective Biodiversity Conservation

Traditional biodiversity conservation approaches paid more attention to habitat carrying capacity and landscape structural connectivity, focusing on habitat area and heterogeneity and the geometric connection of patches, yet largely overlooked the species dispersal process (Schleicher et al. 2011). Subsequent research demonstrated that biodiversity is also influenced by species dispersal capacity in addition to habitat spatial patterns (Kimberley et al. 2021). In fragmented habitats, the species dispersal process determines whether species can colonize adjacent habitat patches and thereby alters regional biodiversity patterns (Vasiliev and Greenwood 2023). Biodiversity is jointly determined by habitat attributes and biological traits (Louis‐Lucas et al. 2025). Effective biodiversity conservation planning therefore requires an integrated perspective that couples landscape pattern and ecological processes through incorporating habitat spatial structure to organismal dispersal behavior. As an innovation, this study parsed the impacts of meadow connectivity on plant diversity by considering plant species dispersal process, demonstrating that functional connectivity explains plant diversity better than structural connectivity. Our findings provide a novel insight into the modern biodiversity conservation, that is, prioritizing the restoration and enhancement of landscape connectivity, particularly functional connectivity, to facilitate species dispersal and successful establishment.

### 4.4 Implications for Habitat Management

In habitat management, environmental protectors typically prioritize patch area, focusing on identifying and protecting large patches, while overlooking landscape connectivity (Zhao et al. 2023). This study provides novel information about the dynamics of meadow connectivity at different water levels, thereby establishing a scientific foundation for meadow habitat management based on landscape connectivity in Poyang Lake. Moreover, conservation practitioners have long been confused about whether to protect all habitat patches broadly or to allocate limited resources toward safeguarding a subset of ecologically critical patches (Lamounier et al. 2024). In large‐scale habitats, comprehensive protection of all habitat patches is infeasible due to their high number and spatially heterogeneous distribution (Zhao et al. 2021). Consequently, prioritizing represents one of the most cost‐effective strategies for habitat conservation (Wang et al. 2016; Wang et al. 2022). This study identified the spatial pattern of each meadow patch and measured the overall connectivity of meadow, which provided an implication for determining the conservation priorities of meadow patches by assessing the differential role of individual patches in maintaining the overall landscape connectivity.

## 5 Conclusions

This study offers a new perspective on landscape connectivity for sustainable biodiversity conservation. We analyzed the impacts of meadow connectivity on plant diversity and its scale effects, and further explored the contrasted explanatory role of meadow structural connectivity and functional connectivity on plant diversity patterns. The results can be concluded as follows:

1. Water level fluctuations exerted a pronounced influence on meadow spatial distribution. On the one hand, the meadow was inundated by water and gradually shrank when the water level rose. On the other hand, the meadow were divided by water and progressively became fragmented when the water level increased. Across the water level gradient, meadow spatial configuration presented four distinct structural types: band‐shaped, circular, fan‐shaped and patchy.
2. When the water level increased, the shape of the patch simplified, the area of the patch shrank, the density of the patch reduced, the aggregation of the patch decreased, and the meadow structural connectivity decreased progressively. Meanwhile, the number of components rose, the probability of connectivity decreased, and the meadow functional connectivity reduced significantly. Moreover, the α diversity of the plant community decreased significantly with rising water levels, whereas β diversity increased progressively.
3. LRM revealed that meadow connectivity exerts scale‐dependent effects on plant diversity. Higher meadow connectivity not only led to greater plant species richness at the landscape scale but also increased plant community similarity at the patch scale. Both meadow structural connectivity and functional connectivity exhibited consistent directional effects on α and β diversity. In addition to patch area and aggregation, plant diversity is strongly associated with propagule dispersal capacity. RDA demonstrated that meadow functional connectivity explains plant diversity patterns better than structural connectivity.

## Author Contributions

**Cheng Zhang:** data curation (equal), investigation (equal), methodology (equal), writing – original draft (equal). **Wenbo Chen:** conceptualization (equal), funding acquisition (equal), supervision (equal), writing – review and editing (equal). **Qiaoqin Liu:** investigation (equal), software (equal), validation (equal), visualization (equal).

## Funding

This work was supported by the National Natural Science Foundation of China (Grant No. 42471120, 42261021) and the Fundamental Research Funds for the Central Universities of University of Emergency Management (Grant No. ZY20210305).

## Conflicts of Interest

The authors declare no conflicts of interest.

## Supporting information


**Figure S1:** Pearson correlation matrix for landscape structural connectivity indices.
**Figure S2:** Pearson correlation matrix for landscape functional connectivity indices.
**Figure S3:** Changes of NC with different dispersal distances in each ecological zone.
**Figure S4:** Linear regression relationships between α diversity and β diversity.
**Table S1:** Calculation formulas and descriptions of landscape connectivity indices.

## Data Availability

The data that support the findings of this study are openly available in Figshare (https://doi.org/10.6084/m9.figshare.33154919).
