# Supplementary material for: Effects of Meadow Connectivity on Plant Diversity in China's Poyang Lake: New Perspective of Effective Biodiversity Conservation
Source: Ecol Evol. 2026 Sep 6;16(9):e74328. doi: 10.1002/ece3.74328 (PMC13546867; doi:10.1002/ece3.74328)
Supplement: Supplementary file 1 [file ECE3-16-e74328-s001.doc]

**Figure S1 |** Pearson correlation matrix for landscape structural connectivity indices

**Figure S2 |** Pearson correlation matrix for landscape functional connectivity indices

**Figure S3 |** Changes of NC with different dispersal distances in each ecological zone

**Figure S4 |** Linear regression relationships between α diversity and β diversity

**Table S1 |** Calculation formulas and descriptions of landscape connectivity indices

| Connectivity index | Formula | Description and interpretation |
| --- | --- | --- |
| LPI | $LPI=\frac{\max(a_{i})}{A}\times100\%$ $a_{i}$: The area of patch $i$; $A$: The total area of the analysed region. | It represents the proportion of the largest patch in the whole landscape. |
| AWMSI | $AWMSI=\sum_{i=1}^{n} \left[(\frac{0.25P_{i}}{\sqrt{a_{i}}})(\frac{a_{i}}{A})\right]$ $n$: Total number of patches in the landscape;$P_{i}$: The perimeter of patch $i$. | It reflects the complexity of patch shape. |
| PAFRAC | $\begin{matrix} PAFRAC= \\ \frac{2}{\frac{N\sum_{i=1}^{m} \sum_{j=1}^{n} \left(\ln P_{ij}\times\ln a_{ij}\right)-\sum_{i=1}^{m} \sum_{j=1}^{n} \ln P_{ij}\times\sum_{i=1}^{m} \sum_{j=1}^{n} \ln a_{ij}}{N\sum_{i=1}^{m} \sum_{j=1}^{n} \ln P_{ij}^{2}-\sum_{i=1}^{m} \sum_{j=1}^{n} \ln P_{ij}}} \end{matrix}$ | It reflect the interference of natural or human activities to landscape pattern. |
| PD | $PD=\frac{n}{A}$ | It reflects the heterogeneity and fragmentation of the whole landscape. |
| ED | $ED=\sum_{i=1}^{n} e_{i}$ $e_{i}$: The boundary length of patch $i$. | It represents the degree to which the landscape is divided. |
| COHESION | $COHESION=\left[1-\frac{\sum_{i=1}^{m} P_{i}}{\sum_{i=1}^{m} P_{i}\sqrt{a_{i}}}\right]\left[1-\frac{1}{\sqrt{A}}\right]^{-1}\times100\%$ | It reflects the aggregation of patches in the landscape. |
| NC | / | Number of components in which functional relation exists between patches. |
| F | $F=\sum_{i=1}^{n} \sum_{j=1,i\neq j}^{n} p_{ij}$ $p_{ij}$: The probability of direct dispersal between patch $i$ and patch $j$. | It reflects the overall level of patch connection. |
| PC | $PC=(\sum_{i=1}^{n} \sum_{j=1}^{n} a_{i}\cdot a_{j}\cdot p_{ij}^{*})/A_{L}^{2}$ $a_{i}$,$a_{j}$: The area of patch $i$ and patch $j$; $p_{ij}^{*}$: The maximum product probability of all paths between patch $i$ and patch $j$. | It reflects the possibility level of patch connection. |
